# Thermodynamic modelling of synthetic communities predicts minimum free energy requirements for sulfate reduction and methanogenesis

**DOI:** 10.1098/rsif.2020.0053

**Published:** 2020-05-06

**Authors:** Hadrien Delattre, Jing Chen, Matthew J. Wade, Orkun S. Soyer

**Affiliations:** 1 School of Life Sciences, University of Warwick, Coventry, UK; 2 School of Engineering, Newcastle University, Newcastle-upon-Tyne NE1 7RU, UK

**Keywords:** microbial interactions, microbial growth models, time-series data, population dynamics, thermodynamic inhibition

## Abstract

Microbial communities are complex dynamical systems harbouring many species interacting together to implement higher-level functions. Among these higher-level functions, conversion of organic matter into simpler building blocks by microbial communities underpins biogeochemical cycles and animal and plant nutrition, and is exploited in biotechnology. A prerequisite to predicting the dynamics and stability of community-mediated metabolic conversions is the development and calibration of appropriate mathematical models. Here, we present a generic, extendable thermodynamic model for community dynamics and calibrate a key parameter of this thermodynamic model, the minimum energy requirement associated with growth-supporting metabolic pathways, using experimental population dynamics data from synthetic communities composed of a sulfate reducer and two methanogens. Our findings show that accounting for thermodynamics is necessary in capturing the experimental population dynamics of these synthetic communities that feature relevant species using low energy growth pathways. Furthermore, they provide the first estimates for minimum energy requirements of methanogenesis (in the range of −30 kJ mol^−1^) and elaborate on previous estimates of lactate fermentation by sulfate reducers (in the range of −30 to −17 kJ mol^−1^ depending on the culture conditions). The open-source nature of the developed model and demonstration of its use for estimating a key thermodynamic parameter should facilitate further thermodynamic modelling of microbial communities.

## 1. Introduction

Microbial communities are found in diverse habitats including the oceans, soil, animal guts and plant roots. The interconnected metabolic activities in these microbial communities underpin the biogeochemical cycles that feed into the Earth's ecosystem [1] and the nutrient cycles that support the growth of animals and plants [2,3]. The same community-level metabolic activities, and in particular anaerobic digestion (AD), are also exploited in biotechnology for water treatment and bioenergy production from organic waste [4]. Thus, the ability to capture microbial growth rates and metabolic activities within microbial communities is identified as an important prerequisite for the predictive modelling of planetary ecosystem dynamics, animal and plant health, and biotechnological waste valorization [4].

Modelling microbial community dynamics is a significant challenge due to the complexity of these systems. Typical communities, for example those found in the human gut or AD reactors, are composed of hundreds to thousands of distinct microbial species [5,6]. The metabolic activities, and hence the growth, of these different species are interlinked to each other through metabolic interactions that resemble ecological ones [7]. This resemblance has motivated the adaptation of simplified ecological models (e.g. Lotka–Volterra models) to the modelling of microbial communities [8]. While these models allow drawing generalized hypotheses about the role of different types of interactions on microbial community stability [9], they do not capture metabolite dynamics, which are shown to be essential for predicting population dynamics [10].

Microbial growth and metabolite dynamics are historically captured by empirical models such as the Monod growth function [11,12]. These growth functions have been used to capture the dynamics of microbial communities, most notably to construct relatively large-scale models describing AD communities, as used in wastewater treatment engineering [13]. These models reduce system complexity by considering functional groups (so-called ‘guilds’), rather than individual species, thereby capturing key metabolic processes and interactions such as polymer degradation, sulfate reduction and methanogenesis [14,15]. The guild-based approach makes it possible to calibrate and test these models against the key metabolites measured in AD reactors, bringing us closer to predict the performance and stability of communities in biotechnological applications. Towards achieving this goal, however, a key limitation has been the inadequacy of Monod-type models to capture microbial metabolic conversions that are at a low energy level, and thus operating close to thermodynamic equilibrium [16,17]. Such ‘thermodynamic inhibition’ of microbial growth and metabolism is highly relevant to AD, as well as soil, sediment and gut communities, where there is commonly a depletion of strong electron acceptors and a shift of metabolism from high energy respiratory pathways to low energy fermentative pathways [18].

To capture thermodynamic inhibition effects, a simple thermodynamic model has been proposed that adjusts a Monod-type growth function with a thermodynamic factor based on the free energy of the growth-supporting metabolic conversion [16,17,19,20]. This approach is further elaborated upon by considering the fact that part of the free energy from a given metabolic conversion must be invested into cellular maintenance and as a metabolic driving force, thus defining a minimal energy threshold for a growth-supporting pathway [21]. Incorporating such a thermodynamic model has allowed studying the basis of observed diversity in microbial communities [22] and making qualitative predictions on population dynamics in microbial communities [23]. A fully quantitative prediction of population and metabolite dynamics, however, requires that these models implement specific, calibrated kinetic and thermodynamic parameters for each of the accounted microbial species and their metabolic conversions.

Kinetic parameters of microbial growth have been collected over decades of research using monocultures grown under defined conditions. In particular, maximal growth rate (*v*_max_), substrate affinity coefficient (*K_s_*) and biomass yield from the substrate (*Y_s/x_*) have been experimentally estimated for individual species that represent common functional groups seen in microbial communities. For some of these kinetic parameters, in particular biomass yield and substrate uptake rate, calibrated methods have been derived that can predict parameters from existing data and first principles approximations [24–26]. The key thermodynamic parameter, namely the minimum energy threshold of different metabolic conversions, however, remains mostly unavailable. Moreover, there has not been any focussed exploration of what kind of experimental measurements can provide sufficiently robust estimations for this parameter. This situation limits the applicability of thermodynamic community models, which are required to fully capture the growth of many functionally relevant species.

Here, we aim to address this gap and develop a generic, readily extendable thermodynamic community model and use it to estimate the minimal free energy parameters from experimental time-series data. The model implements the multiple and distinct growth-supporting metabolic conversions possible in each organism and accounts for their possible thermodynamic limitations. It also accounts for metabolite phase exchanges and system pH. We calibrate the model using experimental data from synthetic communities composed of microbial species that represent key functional groups in AD systems; *Desulfovibrio vulgaris* (*Dv*), a sulfate reducer, *Methanococcus maripaludis* (*Mm*), a hydrogenotrophic methanogen and *Methanosarcina barkeri* (*Mb*), a methanogen capable of acetoclastic methanogenesis. Using daily metabolite measurement from mono-, co- and tri-cultures over a 21-day experiment, we show that the resulting model provides a superior fit to data, compared to a non-thermodynamic model, and that some of the thermodynamic parameters of the model can be calibrated using time-series data. These results show that thermodynamic models are appropriate and are needed to accurately capture metabolite dynamics in microbial communities, but that their full calibration requires a greater breadth of experimental data.

## 2. Material and methods

### 2.1. Overall model description and availability

**Figure 1.**
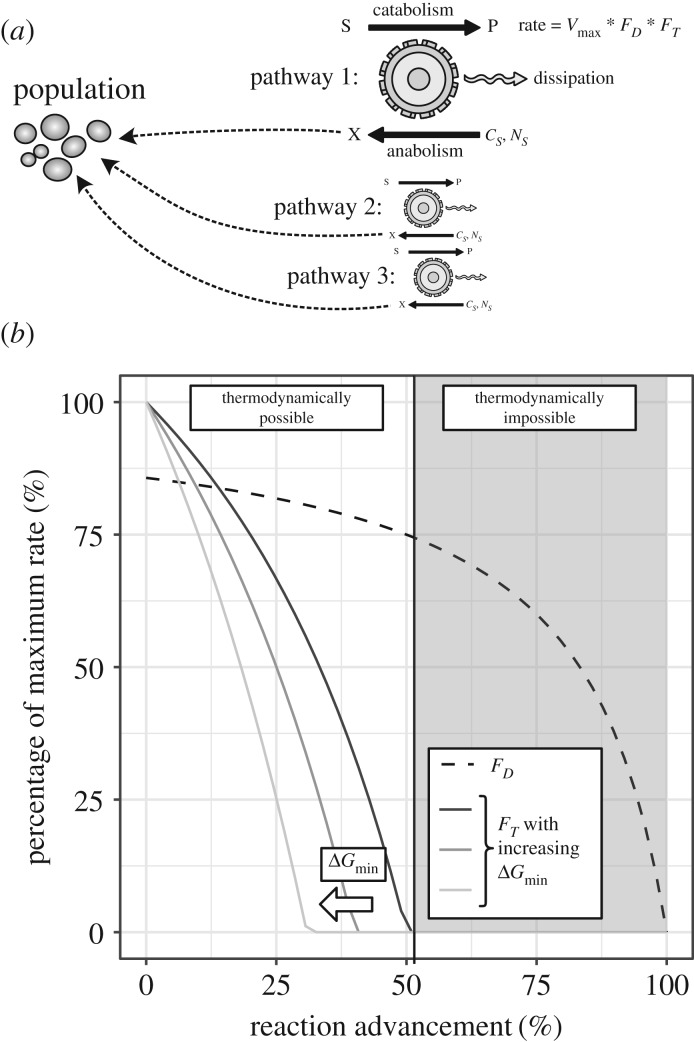
(*a*) Graphical summary of the presented model. Each microbial population is able to catalyse one to multiple different metabolic pathways. Each pathway consists in a catabolic reaction energetically coupled to an anabolic reaction. The rate of the catabolic reaction is given by a constant *v*_max_ multiplied by the *F_D_* and *F_T_* factors, representing enzyme kinetics and thermodynamic constraints, respectively (see Material and methods and equation (2.3)). For each catabolic pathway there is an associated anabolic reaction (which is, for the simulations presented in this article, the same reaction for all catabolic pathways of a given organism). *C_S_* and *N_S_* are, respectively, the carbon source and the nitrogen source for this anabolic reaction. (*b*) Cartoon representation of the value of the *F_D_* and *F_T_* factors as a function of reaction advancement (this representation assumes a simple one-to-one substrate to product stoichiometry). Note that the greater the Δ*G*_min_ parameter (as a negative number), the further left the point where *F_D_* becomes null moves.

The model presented here aims to capture the population and metabolic dynamics within a microbial monoculture or a multi-species community. The model accounts for a set of growth-supporting metabolic pathways that involve either specific metabolites or cellular biomass (figure 1). The chemical speciation of metabolites, as well as their exchange between gas and liquid phases, is accounted for. The medium pH is also simulated based on the set of acid–base reactions that are included. The model is developed in a generic and user-accessible manner, so that growth-supporting reactions, species involved, gas/liquid exchange reactions and acid–base reactions can be supplied by the user without any prerequisite programming skills, and the source-code is extendable by advanced users. The entire model is encoded in an object-oriented software using Python v. 3.4 and the source-code and simulation manual are provided via authors' research website at https://github.com/OSS-Lab/micodymora.

### 2.2. Growth-supporting metabolic pathways

For the presented model, several growth-supporting (i.e. catabolic) and biomass forming (i.e. anabolic) metabolic pathways are considered to be encoded by *Dv*, *Mm* and *Mb* populations, as illustrated in figure 1*a* and listed in table 1. The anabolic (biomass producing) reactions of *Dv*, *Mm* and *Mb* populations are considered to use lactate, carbon dioxide and acetate, respectively, as carbon source. In each of these reactions, biomass is represented as a generic molecule (with chemical formula C_1_H_1.8_O_0.5_N_0.2_), having an associated Gibbs free energy of formation of −67 kJ mol^−1^ [27]. Table 1 lists all growth-supporting catabolic reactions modelled in this work, along with their associated Gibbs free energy (Δ*G*) calculated at a pH of 7.0 and a temperature of 310.15 K with reagents other than protons in their standard state.

**Table 1.** Catabolic and anabolic reactions encoded by each species simulated in the presented model. Reaction Gibbs free energies are also shown, as calculated for pH = 7 (other chemical species' activity assumed to be 1), 1 atm, and 310.15 K.

organism	catabolic pathway(s)	anabolic pathway	metabolic energy
*Dv*	C_3_H_5_O_3_^−^ + 2 H_2_O → C_2_H_3_O_2_^−^ + 2 H_2_(aq) + HCO_3_^−^ + H^+^ Δ*G* = 27.58 kJ mol^−1^	0.35 C_3_H_5_O_3_^−^ + 0.2 NH_4_^+^ + 0.1 H^+^ → C_1_H_1.8_O_0.5_N_0.2_ + 0.05 C_2_H_5_O_2_^−^ + 0.4 H_2_O Δ*G* = 18.11 kJ mol^−1^	Δ*G*_met_ = −265.1 kJ mol^−1^
	C_3_H_5_O_3_^−^ + 0.5 SO_4_^−2^ → C_2_H_5_O_2_^−^ + HCO_3_^−^ + 0.5 H_2_S(aq) Δ*G* = −170.17 kJ mol^−1^		Δ*G*_met_ = −228.2 kJ mol^−1^
	H_2_(aq) + 0.25 SO_4_^−2^ + 0.5 H^+^ → 0.25 H_2_S(aq) + H_2_O Δ*G* = −56.33 kJ mol^−1^		Δ*G*_met_ = −362.1 kJ mol^−1^
*Mm*	0.25 HCO_3_^−^ + H_2_(aq) + 0.25 H^+^ → 0.25 CH_4_(aq) + 0.75 H_2_O Δ*G =* −48.12 kJ mol^−1^	HCO_3_^−^ + 2.1 H_2_(aq) + 0.2 NH_4_^+^ + 0.8 H^+^ → C_1_H_1.8_O_0.5_N_0.2_ + 2.5 H_2_O Δ*G* = −64.73 kJ mol^−1^	Δ*G*_met_ = −876.4 kJ mol^−1^
*Mb*	0.25 HCO_3_^−^ + H_2_(aq) + 0.25 H^+^ → 0.25 CH_4_(aq) + 0.75 H_2_O Δ*G* = −48.12 kJ mol^−1^	0.525 C_2_H_3_O_2_^−^ + 0.2 NH_4_^+^ + 0.275 H^+^ → C_1_H_1.8_O_0.5_N_0.2_ + 0.05 HCO_3_^−^ + 0.4 H_2_O Δ*G* = 28.63 kJ mol^−1^	Δ*G*_met_ = −1059.8 kJ mol^−1^
	C_2_H_3_O_2_^−^ + H_2_O → CH_4_(aq) + HCO_3_^−^ Δ*G* = −14.65 kJ mol^−1^		Δ*G*_met_ = −268.3 kJ mol^−1^

### 2.3. Modelling population and metabolite dynamic

To model population dynamics, we consider the *Dv*, *Mm* and *Mb* populations as implementing catabolic pathways available for each species. The overall dynamics of each population are governed by a differential equation that accounts for all of its catabolic pathways rates, as well as its anabolic biomass production. It is assumed that for a given population, the anabolic reaction has the same formula for each of its catabolic pathways (table 1). To account for the number of times the catabolic reaction has to run per anabolic reaction to close the energy balance of the metabolism, the stoichiometry of each catabolic reaction is multiplied by a dynamic stoichiometry factor (*λ*_cat_, in mol_X_/mol_S_ where X stands for biomass and S for the substrate by which the catabolic pathway's formula is normalized). This factor is computed dynamically from the energetics of catabolic and anabolic reactions, as well as the overall Gibbs energy change during biomass formation

2.1 $$\lambda_{\mathrm{cat}}=\frac{\Delta G_{\mathrm{met},j}-\Delta G_{\mathrm{an}}^{i}}{\Delta G_{\mathrm{cat},j}^{i}},$$

where the indices *i* and *j* range over a given species (e.g. *Dv*) and catabolic pathway (e.g. lactate fermentation), respectively. The $\Delta G_{\mathrm{cat},j}^{i}$ is the Gibbs free energy (in kJ mol_S_ ^−1^), of the associated catabolic pathway *j* in a given species *i*, while Δ*G*_met,*j*_ is the overall Gibbs energy differential of the pathway (further called ‘metabolic energy’). $\Delta G_{\mathrm{an}}^{i}$ is the Gibbs free energy of the anabolic reaction respectively (both in kJ mol_X_ ^−1^) for species *i*. $\Delta G_{\mathrm{an}}^{i}$ has no *j* index because we decided to use the same anabolic reaction stoichiometry for all pathways of a population. The Gibbs free energy of the catabolic and anabolic reactions are calculated dynamically during model simulation from the chemical species concentrations. The metabolic energy is that which is harvested by the population through its catabolism and which is not chemically stored as biomass. It encompasses a wide diversity of processes including heat and entropy emission and cellular maintenance. Its value is estimated based on experimentally measured dissipated energy for different microbial species [28] (table 1). The *λ*_cat_ factor relates to the growth yield associated with a metabolic pathway *j* of a population *i* according to the following relationship:

2.2 $$Y_{i,j}=\frac{1}{\lambda_{\mathrm{cat}}+\gamma_{i,D}},$$

where *γ_i_*_,*D*_ is the stoichiometric coefficient for the electron donor in the anabolic reaction associated with the pathway.

The specific rate (*r_i,j_* in mol_S_ (mol_X_ · h)^−1^) of a catabolic pathway *j* for a given species *i* is given by

2.3 $$r_{i,j}=\left(\nu_{\max,j}^{i}\prod_{k}\frac{\left[S_{k}^{j}\right]}{K_{S_{k}^{j}}+[S_{k}^{j}]}\right)\cdot \left(1-{\text{e}}^{\min\;\left(0,\Delta G_{{\;}_{\mathrm{cat},j}}^{i}-\Delta G_{\min,j}^{i}\right)/R\cdot T}\right),$$

where $\nu_{\max,j}^{i}$ is the maximum catabolic turnover rate expressed in mol_S_ (mol_X_ · h)^−1^ and specific to the pathway *j* and the population *i*, $\left[S_{k}^{j}\right]$ is the concentration of the *k*th limiting substrate of the pathway *j*, $K_{S_{k}^{j}}$ is the half-saturation coefficient for that substrate, $\Delta G_{\mathrm{cat},j}^{i}$ is the Gibbs free energy of the catabolic reaction *j* for species *i*, and $\Delta G_{\min,j}^{i}$ is the minimum energy threshold for that catabolic reaction. This last term ($\Delta G_{\min,j}^{i}$) captures how energy-storing reactions coupled to the catabolic reaction (e.g. conserved moieties regeneration, proton extrusion, etc.) affects its rate. *R* (in kJ (mol · K)^−1^) and *T* (in K) denote the gas constant and system temperature respectively*.* Note that in the main text, we refer to the first and second terms of equation (2.3) as kinetic (*F_D_*) and thermodynamic (*F_T_*) factors, similar to previous presentations in the literature [29]. The kinetic coefficients $\nu_{\max,j}^{i}$ and $K_{S_{k}^{j}}$ are compiled from the literature [30–32] and are listed in electronic supplementary material, table S1.

Using the rate of the catabolic pathway and biomass yield of the anabolic pathway, we can write a differential equation describing the dynamics of the concentration of any chemical *A* according to the catalytic activity of each population

2.4 $$\frac{\text{d}[A]}{\text{d}t}=\underset{i}{\sum }\left([X_{i}]\cdot \underset{j}{\sum }\left(r_{i,j}\cdot \left(\gamma_{i,A}\cdot \frac{1}{\lambda_{\mathrm{cat}}}+\vartheta_{i,j,A}\right)\right)\right),$$

where [*X_i_*] represents the biomass concentration of the *i*th population*, γ_i_*_,*A*_ is the stoichiometric coefficient for chemical *A* in the anabolic reaction of the *i*th population and $\vartheta_{i,j,A}$ is the stoichiometric coefficient for chemical *A* in the *j*th catabolic pathway of the *i*th population. Consequently, the stoichiometry of the different pathways of a given population simply adds up.

The dynamics of the biomass associated with a population *i* obeys essentially the same equation as for the concentration of chemical (equation (2.4)), however, $\vartheta_{i,j,X}$ is always zero because biomass is not produced or consumed by catabolism and *γ_i_*_,*X*_ is always one because the anabolic formula is normalized to the mole of biomass. Additionally, we account for the loss of biomass through death using a linear decay coefficient *k_d_* (1 h^−1^), resulting in the following differential equation for biomass:

2.5 $$\frac{\text{d}[X_{i}]}{dt}=[X_{i}]\cdot \left(\underset{j}{\sum }\left(r_{i,j}\cdot \gamma_{i,X}\cdot \frac{1}{\lambda_{\mathrm{cat}}}\right)-k_{d}\right).$$

We assume in the current model that *k_d_* (=8.33 × 10^−4^ 1 h^−1^) is the same for all species. This value is based on the experimental estimates made on *Desulfovibrio vulgaris* monocultures [32]. Simulations with alternative *k_d_* for *Mb* and *Mm* (between 6.66 × 10^−4^ 1 h^−1^ and 9.99 × 10^−4^ 1 h^−1^) had no significant effect on the results of this manuscript (electronic supplementary material, figure S6).

### 2.4. Modelling of gas/liquid transfer

Some chemicals exist in both the gas and liquid phases. Any of such chemical species, *A*, is accounted for as two separate chemical species *A*(*aq*) and *A*(*g*), respectively. The concentrations of each species are accounted for by moles per litre of their respective phase volume. The transfer dynamics occurring between the two forms is captured through a set of differential equations given by

2.6 $$\frac{\text{d}[A(aq)]}{\text{d}t}=-k_{L}a\cdot \left([A(aq)]-[A(g)]\cdot H_{310.15}\right),$$

and

2.7 $$\frac{\text{d}[A(g)]}{\text{d}t}=k_{L}a\cdot \frac{V_{aq}}{V_{g}}\cdot \left([A(aq)]-[A(g)]\cdot H_{310.15}\right)$$

where *k_L_a* is the mass transfer coefficient of the chemical (in 1 h^−1^) [33], *H*_310.15_ is the Henry constant of the chemical at 310.15 K (and expressed in mol (m^3^ · Pa)^−1^), *V*_aq_ is the volume of the liquid phase and *V_g_* is the volume of the gas phase (both in litres). Henry constants were obtained from the literature [34], and adjusted for a temperature of 310.15 K using the relation between Henry's constant and the solution enthalpy (Δ*H*_sol_) as follows: $H_{310.15}=H_{298.15}\times \exp(\Delta H_{\mathrm{sol}}/R\cdot (1/310.15-1/298.15))$. The list of species that are modelled as distributed between liquid and gas phases, and their associated Henry constants and mass transfer coefficients are listed in electronic supplementary material, table S2.

### 2.5. Modelling of medium pH

At the beginning of each timestep in the integration of the differential equation system (composed of equations (2.4)–(2.5), the pH of the solution is determined. This is done by solving the charge balance of the system using the Brent method [35], while considering the proportion of each ionized species depending on the pH. The acid–base equilibria that are considered and determined at each timestep are listed in electronic supplementary material, table S3, along with the associated pK values.

### 2.6. Model parameters and parameter calibration

The kinetic parameters used in the model are listed in electronic supplementary material, table S1, and are based on experimental estimates given in the literature. Henry constant and mass transfer coefficients (electronic supplementary material, table S2) are compiled from the literature or measured in this study (see below). The thermodynamic parameters are either calculated dynamically (as explained above) or adapted from the literature (table 1). The only parameters of the model that are calibrated against experimental data are the $\Delta G_{\min,j}^{i}$ of the different catabolic pathways. These parameters are calibrated using a recently introduced optimization procedure [36]. This approach has been chosen because it has specifically been proposed in the context of the estimating microbial growth parameters and to circumvent the problem of parameter identifiability [37]. In brief, this approach calibrates multiple parameters (the $\Delta G_{\min,j}^{i}$ of each pathway) against multiple observed variables (experimentally observed lactate, acetate, H_2_(g) and CH_4_(g) concentrations). With this optimization procedure, the parameters are treated in a hierarchical fashion according to two properties; the extent (the number of variables affected upon changing a given parameter) and scale (the level of change in variables induced by changing a given parameter) of their effect. The parameter that produces the strongest effect among the least number of variables is selected first and the experimental time course data of the variables are weighted so that those variables that are most affected by the parameter have more weight in the calculation of the match between model prediction and experimental data during the optimization procedure. The selected parameter is then optimized against the weighted experimental data on variables using the truncated Newton method (here we use the implementation available in the Python v. 3.4, package ‘scipy’). This method minimizes the weighted sum of squared distance between the model predictions and the experimental data on the variables. Once a parameter is optimized in this way, its value is fixed and removed from the list of the parameters to be optimized. The optimization procedure then restarts with the remaining parameters until they have all been optimized and then repeated again for a different set of starting values. The whole process of optimization is repeated until it yields no significant improvement anymore in terms of distance between the model predictions and the experimental data on variables.

### 2.7. Numerical simulations

The model is used to simulate the dynamics of the different populations as well as the key metabolites and system pH. Simulations were run to emulate the actual experiments in terms of run duration and initial starting conditions. The latter was assumed to be an equal biomass distribution among constituting species. To estimate this distribution, total biomass concentration in C-mol l^−1^ was approximated from the experimental OD (at 600 nm) measurements at the start of the experiment (electronic supplementary material, table S4) and using a previously calibrated relationship between OD and biomass using sulfate-reducing bacteria (predominantly *Desulfovibrio vulgaris*) [38]; ln(DW) = 5.12 · OD600 – 4.987, where DW is the dry weight of the cells in g l^−1^. We converted the resulting DW value to 1 C mol^−1^ by dividing it by the molecular weight of the generic molecule used to represent biomass (C_1_H_1.8_O_0.5_N_0.2_, [27]); 24.6 g.C mol^−1^. The resulting biomass concentration was then evenly distributed between the existing populations to create the initial point for simulations.

Simulations were done using the ‘micodymora’ package for Python v. 3.4 (available at; https://github.com/OSS-Lab/micodymora). The calculations performed at each simulation timestep are the following: (i) determine the pH and the speciation of each chemical species (see ‘Modelling of medium pH’), (ii) compute the differential of each chemical species based on their current concentrations for gas/liquid transfer (see ‘Modelling of gas/liquid transfer’) and biochemical reactions (see ‘Modelling of metabolites and populations dynamics') separately, (iii) add the two differential terms together. The integration of the system is done using the ‘odeint’ function of the ‘scipy’ package.

### 2.8. Experimental estimation of *k_L_a* for H_2_, CO_2_ and CH_4_

The *k_L_a* parameter for H_2_, CO_2_ and CH_4_ was estimated based on experimental measurements using the same setup as in our experimental system. The anaerobic medium was prepared as previously described [39], containing 30 mM Na-lactate and 7.5 mM Na_2_SO_4_. Anaerobic culture tubes (Hungate tubes, Chemglass Life Sciences, Vineland, NJ, USA) were prepared with 5 ml medium and 0.1 ml of 100 mM Na_2_S·9H_2_O in each tube, sealed in an anaerobic chamber station (MG500, Don Whitley) and autoclaved. The headspace gas pressure and composition of the tubes were measured using a micro gas-chromatograph (GC) (Agilent 490 micro-GC, Agilent Technologies) and recorded. A gas mixture of H_2_, CO_2_ and CH_4_ was prepared by first flushing two 118 ml serum bottles with 80% H_2_/20% CO_2_ gas mixture for 3 min at 0.5 l min^−1^ flow rate and balancing the final pressure to 1 atm (101 325 Pa). Then, 10 ml of 90% CH_4_/10% CO_2_ gas mixture at 1 atm was injected into each serum bottle using a gas-tight glass syringe (Cadence Science, Inc., Italy). Two millilitres of the resulting gas mixture is injected into each of the prepared Hungate tubes using a gas-tight glass syringe. The tubes were incubated under 37°C for more than 24 h, in order to let the added gas to be equilibrated between the headspace and the aqueous phase. The tubes were then flushed with 100% N_2_ for 2 min at a flow rate of 0.2 l min^−1^ and their headspace pressure brought to 1 atm using sterile needle and filter. The tubes were then returned to the 37°C incubator, and brought out in replicates of three for temporal measurement of headspace gas composition at pre-determined intervals of 0, 1, 2, 4, 8 and 24 h. The resulting temporal gas equilibration data are then used to estimate the *k_L_a* value for H_2_, CO_2_ and CH_4_. Specifically, the *k_L_a* values were obtained by minimizing the sum of squared error between average observed measurements and the integration of the dynamics of gas transfer considered (see equations (2.6) and (2.7)).

### 2.9. Experimental implementation of monocultures and synthetic microbial communities

The three strains of *Dv*, *Mb* and *Mm* and anaerobic medium preparations were done as previously described [39]. In brief, the monocultures of *Dv*, *Mb* and *Mm* were cultivated in 5 ml anaerobic media for 4, 21 and 7 days respectively, to reach their late log phase. These monocultures were all grown at 37°C in the same anaerobic medium base (OSM1.0 media as described in [39]), but with different carbon and energy sources; 30 mM Na-lactate and 10 mM Na_2_SO_4_ for *Dv*, 100 mM Na-acetate for *Mb* and 10 mM Na-pyruvate and 68.4 mM NaCl for *Mm*. For the last species, the headspace is also filled with 80%H_2_/20%CO_2_ gas mixture at a pressure of 2 atm. To create synthetic communities of co- and tri-cultures, we first created stock cultures by taking 2 ml aliquots of each monoculture using sterile needle syringe inside the anaerobic chamber and inoculating these in the combinations of *Dv–Mb*, *Dv–Mm* and *Dv–Mb–Mm* into different serum bottles, which contained 50 ml OSM1.0 medium with 30 mM Na-lactate and 7.5 mM Na_2_SO_4_. The inoculated serum bottles were placed in a 37°C incubator for 21 days. At the end of this period, 17.5 ml cultures of different combinations from the incubated serum bottles were transferred into 500 ml anaerobic Duran bottles containing 350 ml of the above medium. The Duran bottles were linked to a Micro-GC (Agilent 490 micro-GC, Agilent Technologies) for continuous monitoring of the methane production over two weeks. The active methanogenic communities in all combinations were confirmed in this way and the cultures were considered and used as the stock cultures for the following step. Five millilitres of the stock cultures from each combination were extracted inside the anaerobic chamber, mixed separately with 5 ml fresh anaerobic medium OSM1.0 with 30 mM Na-lactate and 7.5 mM Na_2_SO_4_ in Hungate tubes, and incubated at 37°C for 7 days. These cultures formed the inocula for the following time-series experiment.

To measure temporal dynamics of co- and tri-cultures, as well as *Dv* monoculture, we designed a time-series experiment that involved starting a large number of replicate tubes and terminating a set of this large batch at different time points for gas and metabolite measurements. In total, 273 anaerobic Hungate tubes were prepared to collect data for 21 time points. Each tube contained 5 ml OSM1.0 medium with 30 mM Na-lactate and 7.5 mM Na_2_SO_4_. According to the full reaction of sulfate reduction by *Dv* in table 1, 7.5 mM sulfate should allow *Dv* to convert 15 mM lactate fully, while the conversion of the other 15 mM lactate would rely on *Dv*'s other less thermodynamically favourable pathways. The tubes were numbered individually and separated into 21 batches. Each batch contains 13 tubes, of which one tube was used as a blank control and three replicate tubes were used each for the four cultures: *Dv*–*Mb*, *Dv*–*Mm*, *Dv*–*Mb*–*Mm* and *Dv*, respectively. The tubes were inoculated with the respective cultures using the stock cultures described above, and following the tube and batch numbers. The initial optical density (OD) at 600 nm and headspace pressure were recorded for each tube using a spectrophotometer (Spectronic 200E, Thermo Scientific) and a needle pressure gauge (ASHCROFT 310, USA). All tubes were incubated at 37°C. Over the following 21 days, 13 tubes of one batch were terminated on each single day to measure their OD at 600 nm, pH (Mettler Toledo M300, Columbus, OH, USA), gas pressure (ASHCROFT 310, USA), gas composition using Micro-GC (Agilent 490 micro-GC, Agilent Technologies) and the lactate, acetate, pyruvate and sulfate concentrations using ion chromatography (Dionex ICS-5000^+^ DP, Thermo Scientific) as described previously [39].

## 3. Results and discussion

To develop and calibrate a thermodynamic model of microbial growth and metabolite dynamics in a community context, we focus here on defined anaerobic synthetic communities. In particular, we use a recently developed experimental model system for studying syntrophic interactions among sulfate reducers and methanogens [39,40], which make up a key part of anaerobic microbial communities found in AD reactors and freshwater and estuary sediments. The studied synthetic systems are composed of a representative sulfate reducer (*Desulfovibrio vulgaris*, *Dv*), and two different methanogens representing hydrogenotrophic (*Methanococcus maripaludis*, *Mm*) and hydrogeno/acetotrophic (*Methanosarcina barkeri*, *Mb*) methanogenesis pathways (see Material and methods and figure 1). We have collected here data on metabolite dynamics over a three-week period from *Dv* monocultures, *Dv*–*Mm* and *Dv*–*Mb* co-cultures and *Dv*–*Mm*–*Mb* tri-cultures under specific media conditions (see Material and methods).

### 3.1. A comprehensive and generic thermodynamic model of community dynamics

To capture community and metabolite dynamics, we developed a comprehensive and expandable thermodynamic model that also accounts for metabolite phase exchanges and medium pH (see Material and methods). As is common for many microbes found in microbial communities, the species composing the studied synthetic communities can catalyse multiple, distinct metabolic pathways, sometimes starting from the same substrate. To account for these different metabolic activities of each species, we considered that each population can use any number of pathways at once, as previously described [32]. Each pathway consists of a catabolic (energy harvesting) reaction and an anabolic (biomass synthesis) reaction (figure 1). The number of anabolic turnovers per catabolic turnover is then determined dynamically based on the energy flux provided by the catabolic reaction and on the cost of biomass production (see equation (2.1)). The latter is computed accounting for biomass synthesis cost and a constant ‘metabolic’ cost per amount of biomass, based on recent estimations [28]. Therefore, this model implements a dynamic biomass yield based on energy considerations. By lumping all the catabolic energy that is not incorporated into biomass as a constant ‘metabolic’ term, we implicitly assume that maintenance, which is then lumped with other forms of energy dissipation, is constant. While more sophisticated dynamic representations of maintenance exist [41,42], these approaches would add more complexity to the current model, which aims to assess how a simpler, more parsimonious thermodynamic modelling approach can capture experimental population dynamics.

The specific rate of the catabolic reaction is determined by the product of a kinetic factor (*F_D_*), expressing enzyme kinetics and a thermodynamic factor (*F_T_*), expressing the limitations arising from thermodynamic constraints (figure 1 and equation (2.3)). *F_T_* accounts for the energetic feasibility of the growth-supporting pathway, as well as a minimal energy requirement (Δ*G*_min_). The Δ*G*_min_ represents the concept that cells must invest some of the energy associated with each catabolic into a metabolic driving force to run that reaction, as well as into maintaining cell viability. It is assumed that such an energy investment is pathway specific and its value can be estimated from population dynamics data, as attempted here. The resulting model is parameterized for kinetic rates using available estimates for *Dv*, *Mm* and *Mb* (see electronic supplementary material, table S1). After this parameterization, the only unknown parameters in the system are the Δ*G*_min_, which we have estimated here from the data, and some of the metabolite phase exchange constants, which we have determined experimentally. This model is developed in a generic manner allowing its expansion to include additional species and metabolic conversions. This makes it adaptable to other monocultures and natural or synthetic communities (see Material and methods).

One key feature of this generic thermodynamic model is that it implements dynamic metabolic stoichiometry through a variable yield term. The use of variable yield adjusted to close the energy balance of metabolism has indeed been advocated as a necessary feature to represent anaerobic metabolism dynamics [43,44] and implemented in previous dynamic models describing the microbial community in terms of ‘functional guilds’, i.e. assigning each possible pathway to one specific population [23,45]. The presented model distinguishes itself from those precedents as it models the growth of phylogenetically defined populations able to catalyse multiple pathways; in other words, multiple energy gradients in a culture medium can benefit the same population. Moreover, determining the yield from physical quantities (energy gradients) reduces the amount of parameters to calibrate and thus improves the identifiability of the model's parameters [36]. Another feature of the model is the implementation of chemical speciation in order to get a more realistic representation of the pH dynamics and the chemical concentrations during the simulations. This point, while being a rather technical one, is important especially when a dissolved species involved in a metabolic pathway has also a counterpart in the gas phase (e.g. hydrogen). In such cases, the presented model accounts for the concentration of the dissolved species in the mass action ratio of a growth-supporting metabolic pathway when determining the Gibbs free energy of that pathway.

### 3.2. A thermodynamic inhibition model is required to correctly capture community metabolite dynamics

**Figure 2.**
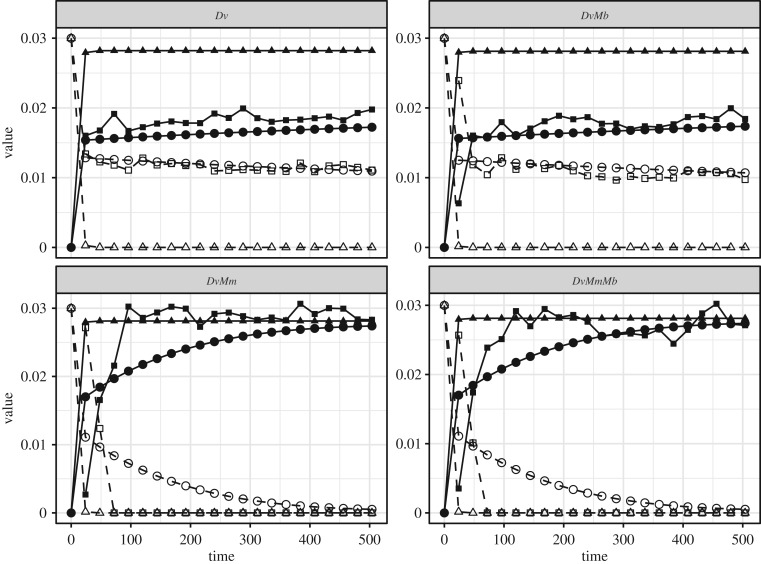
Concentration (mol l^−1^) of acetate (solid line and filled shapes) and lactate (dashed line and empty shapes) over time (h) in the four different culture cases (*Dv*, *DvMm*, *DvMb*, *DvMmMb*) as measured in experiments (square) versus simulated by the model (triangle or circle). Squares represent the median of the three experimental replicates, triangles represent simulations done without *F_T_* factor (see equation (2.3)), circles represent simulations done with the *F_T_* factor and using Δ*G*_min_ parameters obtained from calibration of the experimental data.

The *F_T_* factor in the presented model introduces a mechanism for thermodynamic inhibition in the model, as done previously [17]. The same model without this factor could be considered as a purely ‘forward reaction kinetics model’ that considers catabolism as an irreversible process, limited only by substrate concentration [16,22] (figure 1 for illustration). We evaluate these two types of models in their ability to capture metabolite dynamics in our synthetic communities. As explained above, the model without the thermodynamic factor is nested in the presented model in the sense that it results from setting the *F_T_* term to one in equation (2.3). When we do so and use previously determined kinetic parameters (listed in electronic supplementary material, table S1), we can apply the resulting model without thermodynamic inhibition to the experimental data. We find that such a model is not able to explain the observed experimental results (figure 2). In particular, this model suggests the full conversion of lactate in all culture conditions, while we do find significant lactate remaining in both *Dv* monoculture and *DvMb* co-culture. This qualitative mismatch between an experiment and a non-thermodynamic inhibition model is directly a result of the structure of this model. Such a model cannot account for the low energy of lactate fermentation in the absence of sulfate, and therefore incorrectly predicts that *Dv* can consume all of the lactate. Note that this result would not change if we allow fitting of kinetic parameters in the kinetic model, as there is no mechanism in the model to allow for ‘shutting down’ of lactate consumption. The thermodynamic model, instead, allows for such a mechanism through the *F_T_* term in equation (2.3), and as discussed in the next section, this feature allows it to better capture the experimental data.

### 3.3. Calibration of thermodynamic model allows prediction of minimal energy investments during growth with different metabolic pathways

The thermodynamic model allows better capturing of the metabolite dynamics, as shown in figure 2. In this case, the model features additional Δ*G*_min_ parameters associated with the *F_T_* term in equation (2.3). As described above, this parameter captures the associated energy investment from each catabolic reaction into ‘running’ that reaction as well as the amount of energy harvested by the cell (as ATP or membrane gradient [18,20,43,46]). In order to determine this parameter for each of the possible metabolic pathways that can be used by *Dv*, *Mm* and *Mb*, we calibrated the model using an iterative fitting procedure described recently [36] (see Material and methods). The calibration process starts with an initial Δ*G*_min_ value of −40 kJ mol^−1^, based on values from various sources for the Gibbs energy of formation of ATP [17,47], and is applied using all possible combinations of the experimentally observed dynamics to result in the predicted Δ*G*_min_ values for each of the growth-supporting metabolic pathways (table 2).

**Table 2.** Calibrated Δ*G*_min_ values for the different growth-supporting metabolic pathways modelled in this study. All values are in kJ mol^−1^. The different rows indicate the experimental data used for the calibration. Additional results from using combinations of experimental data are provided in the electronic supplementary material, figures S1–S4.

culture	calibration variable	lactate fermentation	*Mm* hydrogenotrophic methanogenesis	*Mb* hydrogenotrophic methanogenesis	*Mb* acetoclastic methanogenesis
*Dv*	H_2_(g)	−32			
	Ac	−27			
*DvMm*	H_2_(g)	<−35	>−25		
	Ac	−17			
	CH_4_(g)		>−25		
*DvMb*	H_2_(g)	−32			
	Ac	−27			
	CH_4_(g)			>−25	>−40
*DvMmMb*	H_2_(g)	<−35	<−25	>−20	
	Ac	−17	<−40	<−40	
	CH_4_(g)		<−30	>−30	>−40

**Figure 3.**
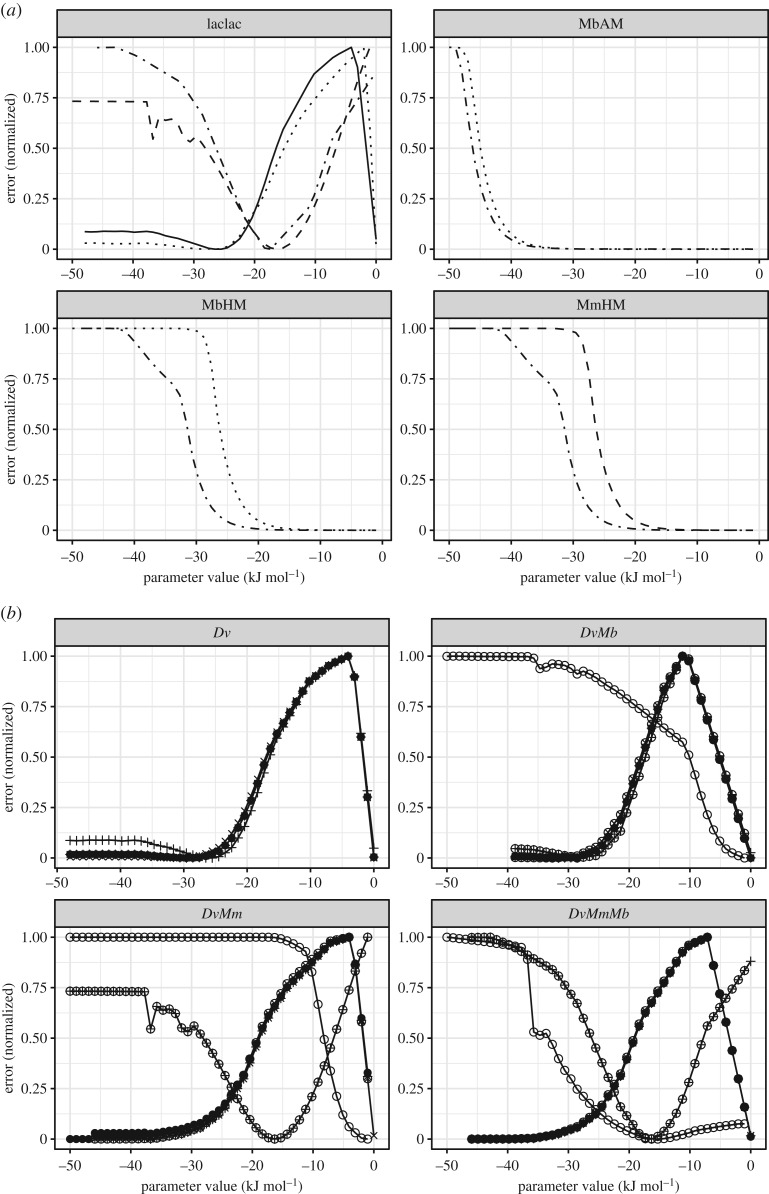
Sum of squared differences (error) between the experimentally observed variable(s) and the model prediction (*y*-axis) as a function of the value of the Δ*G*_min_ of various pathways (*x*-axis). (*a*) Normalized error between experimentally observed versus predicted metabolite concentrations against the value of Δ*G*_min_ for various pathways; lactate fermentation by *Dv* (laclac), acetoclastic methanogenesis by *Mb* (AM), hydrogenotrophic methanogenesis by *Mb* (HM) and hydrogenotrophic methanogenesis by *Mm* (HM). The normalized error values shown are those based on experimentally observed acetate concentration for the laclac case and experimentally observed methane concentration for all other cases. Normalization was done for each case separately using its own maxima. Results for the different cultures are indicated with the line properties; solid line for *Dv*, dashed line for *DvMm*, dotted line for *DvMb*, dashed-dotted line for *DvMmMb*. (*b*) Normalized error, as a function of the estimated value of the Δ*G*_min_ of the lactate fermentation pathway. Normalization was done for each case separately using its own maxima. Each tile corresponds to the results from a different culture case as shown in panel heading. For the different lines shown, the error is computed on different experimentally observed metabolite concentrations as follows; acetate (plus), H_2_(g) (cross), CH_4_(g) (circle), acetate and H_2_(g)- (plus and cross), H_2_(g) and CH_4_(g) (circle and plus), acetate and CH_4_(g) (circle and cross), and H_2_(g), acetate and CH_4_(g) (black filled circles).

After a set of parameters was determined by the calibration procedure for each combination of observed variables, a parametric sweep was performed to determine whether the obtained values correspond to an optimum that minimizes the distance between simulation and observation. We assess this by plotting an error function (see Material and methods) for each calibrated parameter value (figure 3 and electronic supplementary material, figures S1–S4). The shape of the error function around the calibrated values of each parameter indicates that the Δ*G*_min_ of the lactate fermentation pathway has a clear optimum regarding the output variables considered (acetate and H_2_ in gas phase), and lies between −30 and −15 kJ mol^−1^ (figure 3*a*). There seems to be an alternative optimum at 0 kJ mol^−1^ as well, and while using this Δ*G*_min_ value for simulations can result in a good fit of experimental data (electronic supplementary material, figure S5), we do not consider this optimum due to its biological infeasibility (it would imply that none of the catabolic energy is stored into conserved moieties or membrane gradient). The Δ*G*_min_ of the hydrogenic and acetoclastic methanogenesis pathways cannot be given an exact estimate but rather boundaries; less negative than −20 kJ mol^−1^ for hydrogenic methanogenesis and less negative than −40 kJ mol^−1^ for acetoclastic methanogenesis (figure 3*a*). The Δ*G*_min_ parameters for *Dv*'s sulfate respiration pathways could not be calibrated with the present experimental data, presumably because sulfate respiration occurs relatively quickly compared to the time-resolution of the available experimental data.

### 3.4. Predicted Gibbs free energy thresholds for lactate fermentation depends on the co-culture/community context

Interestingly, we find that the lactate fermentation pathway has well-defined and distinct Δ*G*_min_ optima depending on the culture conditions; in the *Dv* and *DvMb* cultures, the optimum is around −30 or −25 kJ mol^−1^ (depending on whether the calibration is based on acetate or H_2_(g)), while in the *DvMm* and *DvMmMb* cultures, the optimum is clearly around −17 kJ mol^−1^ (figure 3*a*)*.* Consequently, simulating the *Dv* culture with the Δ*G*_min_ calibrated on the *DvMm* and *DvMmMb* cultures (−17 kJ mol^−1^) and simulating the *DvMmMb* co-culture with the Δ*G*_min_ calibrated on the *Dv* and *DvMb* cultures (−30 kJ mol^−1^) results in poor fit of experimental data (electronic supplementary material, figure S5). This finding suggests that the Δ*G*_min_, i.e. the amount of energy harvested by the cell from a pathway as ATP or membrane gradient is not a constant. Rather, our observations suggest that it depends on the context in which the pathway is operating, and that some interactions with other organisms may elicit changes in the way cells tune their metabolism which may result in significant modification of their Gibbs free energy thresholds.

As far as we are aware, the estimation of the Δ*G*_min_ parameter has been attempted before in only few studies [20,32]. The minimal free energy for the three metabolic pathways of *Dv* (lactate fermentation and sulfate respiration with lactate or hydrogen) has been calibrated against experimental data only from monocultures grown in the presence of sulfate, and using a model similar to that presented here [32]. The experimental design in that study was different, using solely a monoculture, rather than monoculture and communities as we do here, sampling at shorter time intervals and using higher sulfate concentration than this study. Perhaps due to such differences, the estimated value from that study for lactate fermentation was −39.5 kJ mol^−1^, relatively higher, i.e. more energy investment required as driving force and into maintenance, than found here. Possibly due to its use of higher sulfate concentration and shorter sampling intervals, that study was able to estimate the Δ*G*_min_ for both lactate and H_2_ respiration on sulfate, as −44.66 kJ mol^−1^. It should be noted that the model used by that study is different from the model used here, in that it uses gas partial pressure when calculating reaction free energies and has taken a static approach to model biomass yield. A theoretical study aimed at estimating energetic parameters for several metabolic pathways, including methanogenesis, using existing data [48], but it used a notion of minimum energy threshold that requires assumptions about the underlying metabolic reactions. The final minimum energy threshold is then expressed in terms of ATP molecules produced per metabolic pathway turnover. The resulting predictions from that study cannot be directly translated into a minimum energy threshold if we assume that the Gibbs free energy carried by an ATP molecule varies dynamically with the state of the cell's ATP pool, and therefore cannot be compared directly to the results presented here. Previous models of microbial growth accounting for a minimum energy threshold have been proposed, where the threshold was based on theoretical considerations rather than calibrated from experimental values. Thus, both Kleerebezem & Stams [43] and González-Cabaleiro *et al*. [49] considered the energy of proton extrusion, that is, roughly −23 kJ mol^−1^, as observed by Schink [18]. The values we calibrate are sometimes below this threshold, however, they are in line with the energy ranges reported for co-cultures of *Desulfovibrio vulgaris* with various syntrophs [46].

It is interesting to note that when a parameter sweep shows the existence of an optimal value or range, these depend on the experimental variable and culture used for calibration (table 2, figure 3*b*). There are two possible explanations for this observation. Firstly, there may be metabolic pathways being catalysed by the populations in the different experimental batches that are not represented by the model. If such pathways involve a specific metabolite, then calibrations performed on that metabolite versus some other metabolite might differ. Such an explanation, while theoretically possible, does not fit with the fact that the presented model accounts for all key pathways known to be catalysed by *Dv*, *Mm* and *Mb*. The second possible explanation is that the minimum energy threshold of a given metabolic pathway depends on the experimental conditions. Indeed, the concept of a minimum energy threshold for growth aims to conceptualize energy invested as the metabolic driving force as well as cellular maintenance. Both these energetic investments are expected to be a function of culture and cellular conditions, including specific cellular details such as Mg^2+^ concentration [50]). The minimum energy threshold of a growth-supporting pathway is then expected to be dynamic. However, accurately predicting those dynamic variations would require implementation of a detailed model of the populations' metabolic networks and cellular states.

## 4. Conclusion

Here, we have developed and presented a generic thermodynamic model for capturing population and metabolite dynamics in a microbial community. The model implements specific features that have been proposed and advocated over the last two decades [22,28,33,41] by introducing factors based on first principles (thermodynamic limitation of reaction rate [51]). As such, it overcomes the limitations of modelling approaches solely based on Michaelis–Menten-type kinetics and empirically calibrated product inhibitions (such as with the ADM1 model [13]). We applied this model to capture the dynamics of synthetic communities composed of a sulfate reducer and two methanogens. We also used the model to attempt an estimation of the minimum energy thresholds of the different growth-supporting metabolic pathways found in these organisms, sulfate respiration, lactate fermentation and hydrogenotrophic and acetoclastic methanogenesis. Our findings show that the presented model, while simple, is indeed able to capture some of the thermodynamic limitations occurring in the observed dynamics. Further, the use of the model on experimental data allows for the prediction of the minimum energy requirements for sulfate fermentation and hydrogenotrophic and acetoclastic methanogenesis in a culture context-dependent manner.

Our calibration results also shed light on the limitations of the thermodynamic approach employed. In particular, despite improvements over a non-thermodynamic model, the presented model was not able to fully capture experimental metabolite and population dynamics and its combination with experimental data did not allow the minimal free energies for all modelled pathways to be determined precisely. These limitations might be inherent in the structure of the model or in the experimental design used here, or a combination of the two. The latter can be addressed particularly by collecting more and higher resolution temporal data from similar experimental systems. The former will probably require increasing the complexity of the presented model. In particular, considering minimum energy thresholds as a constant feature of the system may be too simplistic and might instead require the inclusion of elements of metabolic pathway dynamics within the bacterial growth models.

Thermodynamic constraints that we have endeavoured to predict here are one of the few phenomena that can be safely assumed to apply for all growth-supporting metabolic pathways. A sound basis for the description of this fundamental constraint applying to metabolic dynamics is thus necessary before attempting to assess and calibrate the extent of higher-order phenomena such as genetic regulation or resource allocation. While further dedicated experiments and more complex models are necessary to improve the accuracy of dynamics predictions, the presented work provides a step towards this aim. The presented model expands previous efforts of minimal energy estimates from monocultures [32] and combines several recently proposed model features such as dynamic growth yield [22,41] with additional features such as modelling of multiple pathways within individual species, phase exchange dynamics and pH. As such, its further use and assessment will facilitate thermodynamic modelling of microbial community dynamics and estimation of energetic parameters, helping the development of more predictive microbial community dynamics models.

## Supplementary Material



## Data accessibility

All relevant data are presented in the main and electronic supplementary material. Experimental data are available from the authors in electronic format upon request. Models and their source-code are available on O.S.S research group git hub repository pages.
