# Supplementary material for: Thermodynamic modelling of synthetic communities predicts minimum free energy requirements for sulfate reduction and methanogenesis
Source: J R Soc Interface. 2020 May 6;17(166):20200053. doi: 10.1098/rsif.2020.0053 (PMC7276542; doi:10.1098/rsif.2020.0053)
Supplement: Supplementary file 1 [file rsif20200053supp1.docx]

**Supplementary material**

**for**

**Thermodynamic modelling of synthetic communities predicts minimum free energy requirements for sulfate reduction and methanogenesis.**

Hadrien Delattre^1,*,^^, Jing Chen^1,*,#^, Matthew Wade^2^, Orkun S Soyer ^1,^^

| **Organism** | **Metabolism** | **Parameter** | **Value** | **Citation** |
| --- | --- | --- | --- | --- |
| *Dv* | Lactate fermentation | *v_max_* | 1.03 mol_lactate_/C-molX/h | [1] |
| *Dv* | Lactate fermentation | *K_S_ (Lactate)* | 29e-3 mol/L | [1] |
| *Dv* | Lactate respiration | *v_max_* | 1.88 mol_lactate_/C-molX/h | [1] |
| *Dv* | Lactate respiration | *K_S_ (Lactate)* | 29e-3 mol/L | [1] |
| *Dv* | Lactate respiration | *K_S_ (SO_4_^‑2^)* | 210e-6 mol/L | [1] |
| *Dv* | H_2_ respiration | *v_max_* | 3.392 mol_H2_/C-molX/h | [1] |
| *Dv* | H_2_ respiration | *K_S_ (SO_4_^‑2^)* | 210e-6 mol/L | [1] |
| *Dv* | H_2_ respiration | *K_S_ (H_2_,aq)* | 1.4e-6 mol/L | [2] |
| *Mm* | Hydrogenotrophic methanogenesis | *v_max_* | 8.49e-4 mol_H2_/C-mol_X_/h | [2] |
| *Mm* | Hydrogenotrophic methanogenesis | *K_s_* (H_2,aq_) | 6.15e‑6 mol/L | [2] |
| *Mb* | Acetoclastic methanogenesis | *v_max_* | 1.37e-4 mol_Acetate_/C-mol_X_/h | [3] |
| *Mb* | Acetoclastic methanogenesis | *K_s_* (Acetate) | 4.5e-3 mol/L | [3] |
| *Mb* | Hydrogenotrophic methanogenesis | *v_max_* | 1.05e-3 mol_H2_/C-mol_X_/h | [3] |
| *Mb* | Hydrogenotrophic methanogenesis | *K_s_* (H_2,aq_) | 9.5e-6 mol/L | [3] |

**Table S1:** Value and literature source for the parameters of the enzymatic kinetics of the modelled pathways. Values originally expressed per gram of biomass were converted to C­‑mol of biomass by assuming C_5_H_7_O_2_N formula for biomass [4] (Hoover and Porges, 1952) and a molecular weight of 22.62 g/C‑mol. The K_S_ value for H_2_ in Dv’s H_2_ respiration pathway is the arithmetic mean between values reported by Robinson & Tiedje for various Desulfovibrio strains [2]. The K_S_ for hydrogen in Mm’s H_2_ respiration pathway is the arithmetic mean between the values reported by Robinson & Tiedje for various methanogen strains [2].

| **Chemical** | **Henry’s Constant at 298K (mol/(m^3^.Pa)** | **ΔH_sol_/R (K)** | ***k_L_a* (1/h)** | **Citation** |
| --- | --- | --- | --- | --- |
| CO_2_ | 3.4e-4 | 2400 | 1.10e-1 | This study |
| H_2_ | 7.8e-6 | 500 | 1.33e-1 | This study |
| CH_4_ | 1.43e-5 | 1600 | 1.21e-1 | Estimated as geometric mean between the *k_L_a* values for CO_2_ and H_2_ obtained in this study |
| H_2_S | 1.0e-3 | 2100 | 8.33e-2 | [1] |
| NH_3_ | 5.9e-1 | 4200 | 1.21e-1 | Estimated as geometric mean between the *k_L_a* values for CO_2_ and H_2_ obtained in this study |

**Table S2**: Henry’s constants and gas/liquid transfer rate parameters used for the simulations.

| **Chemical** | **pK constant(s)** |
| --- | --- |
| C_2_H_4_O_2_ ⇌ C_2_H_3_O_2_^‑^ | 4.38 |
| C_3_H_6_O_3_ ⇌ C_3_H_5_O_3_^‑^ | 3.78 |
| H_3_PO_4_ ⇌ H_2_PO_4_^‑^ ⇌ HPO_4_^‑2^ ⇌ PO_4_^‑3^ | 1.79; 6.95; 12.89 |
| CO_2_(aq) ⇌ HCO_3_^‑^ ⇌ CO_3_^‑2^ | 6.35; 10.33 |
| H_2_S(aq) ⇌ HS^‑^ | 7.00 |
| HSO_4_^‑^ ⇌ SO_4_^‑2^ | 1.99 |
| NH_4_^+^ ⇌ NH_3_(aq) | 9.25 |

**Table S3:** Acid/base equilibria considered in the simulations and their respective pK constant (at 310.15 K)

| **Case** | **OD600 measurement at day 0** | | | **Average OD600** | **Biomass concentration estimate (C-mol/L)** |
| --- | --- | --- | --- | --- | --- |
|  | **Replicate 1** | **Replicate 2** | **Replicate 3** |  |  |
| ***Dv*** | 0.379 | 0.375 | 0.411 | 0.388 | 2.02e-3 |
| ***DvMm*** | 0.028 | 0.027 | 0.021 | 0.025 | 3.15e-4 |
| ***DvMb*** | 0.110 | 0.115 | 0.158 | 0.128 | 5.34e-4 |
| ***DvMmMb*** | 0.05 | 0.038 | 0.053 | 0.047 | 5.53e-4 |

**Table S4:** Initial biomass concentration in each experimental case

| **Parameter** | **Units** | **Specific to** |
| --- | --- | --- |
| Maximum growth rate | mol/C‑mol_X_/h | Pathway |
| Affinity coefficient (K_S_) | mol/L | Pathway |
| Minimum energy threshold (ΔG_min_) | kJ/mol | Pathway |
| Energy barrier (ΔG_met_) | kJ/C‑molX | Pathway |
| Anabolism formula |  | Population |
| Biomass formula |  | Population |
| Decay rate | 1/h | Population |

**Table S5:** Summary of all the computational parameters needed to run the micodymora program to simulate a single microbial population.


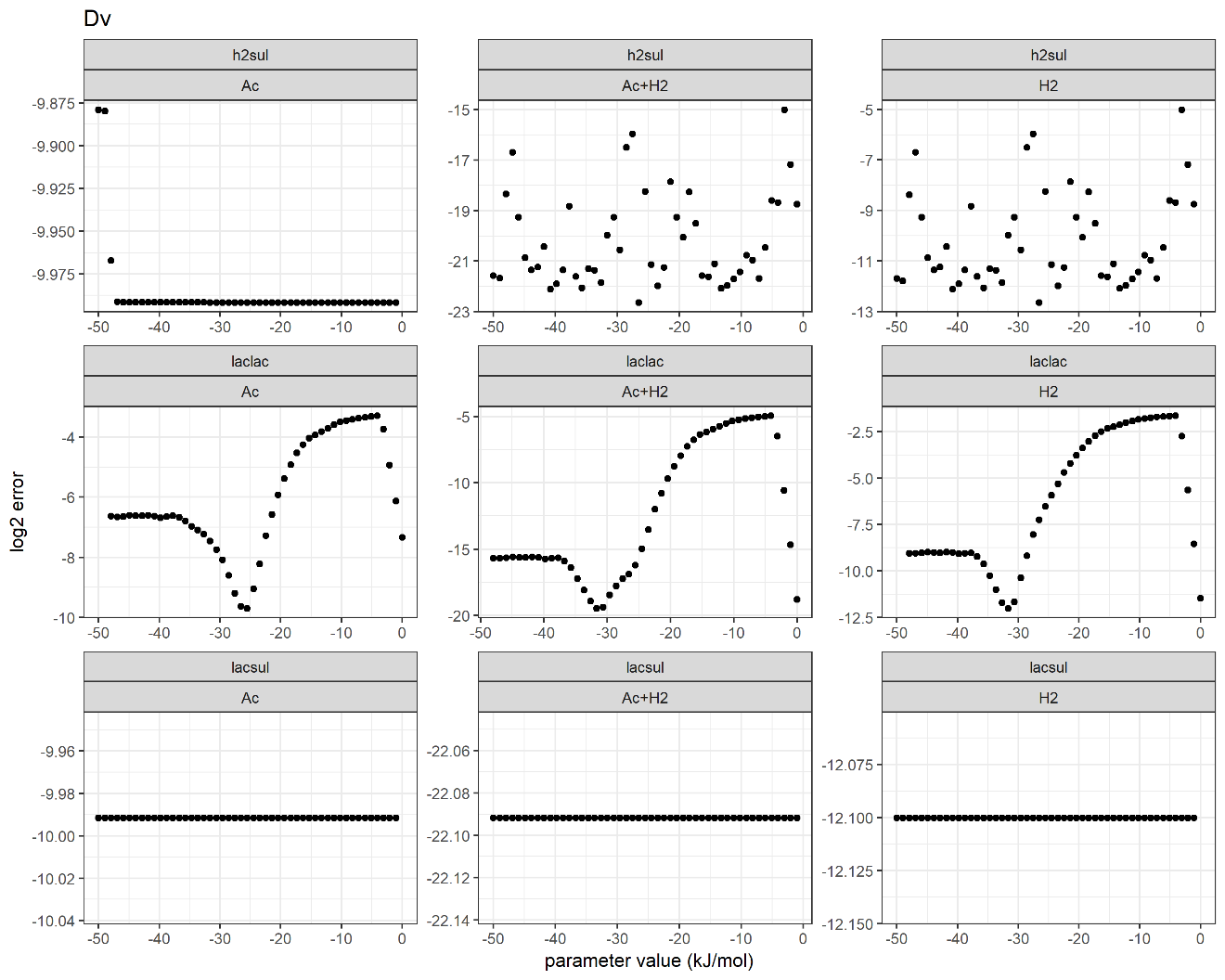


**Figure S1:** Log of the sum of squared differences (error) between the experimentally observed variable(s) and the model prediction. Results are shown for the *Dv* monoculture and as a function of the value of the *ΔG_min_* parameter (kJ/mol) of various pathways and using specific experimental dataset (as indicated on each tile). Notation used for pathways is; laclac: lactate fermentation, lacsul: lactate respiration on sulfate, h2sul: hydrogen respiration on sulfate.


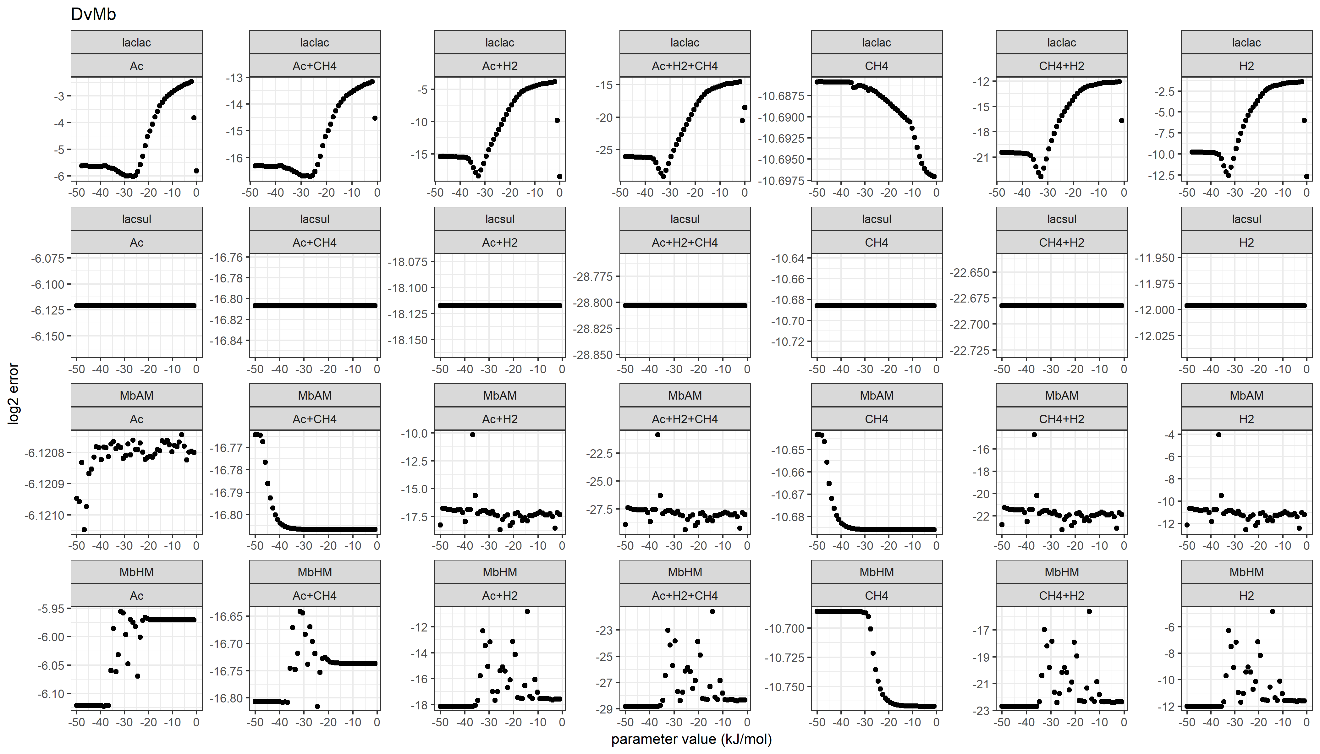


**Figure S2:** Log of the sum of squared differences (error) between the experimentally observed variable(s) and the model prediction. Results are shown for the *DvMb* coculture and as a function of the value of the *ΔG_min_* parameter (kJ/mol) of various pathways and using specific experimental dataset (as indicated on each tile). Notation used for pathways is; laclac: lactate fermentation by *Dv*, lacsul: lactate respiration on sulfate by *Dv*, MbAM: acetoclastic methanogenesis by *Mb*, MbHM: hydrogenotrophic methanogenesis by *Mb*.


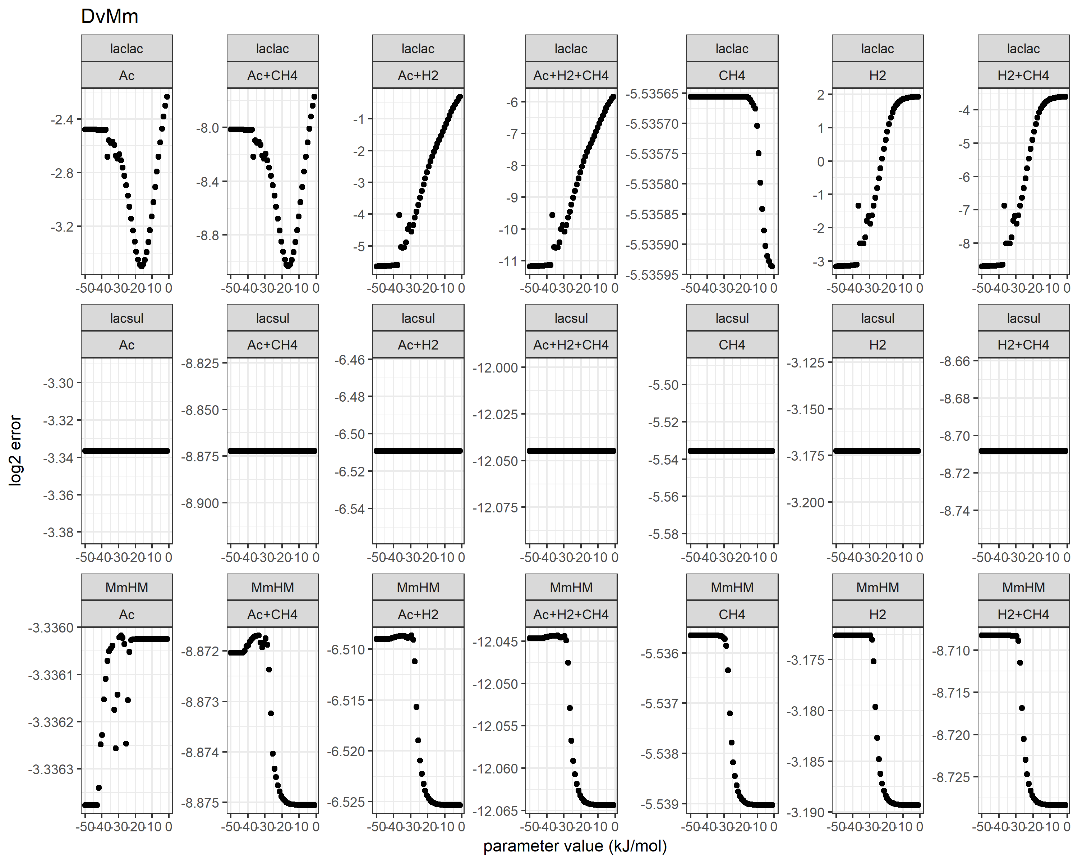


**Figure S3:** Log of the sum of squared differences (error) between the experimentally observed variable(s) and the model prediction. Results are show for the *DvMm* coculture as a function of the value of the *ΔG_min_* parameter (kJ/mol) of various pathways and using specific experimental dataset (as indicated on each tile). Notation used for pathways is; laclac: lactate fermentation by *Dv*, lacsul: lactate respiration on sulfate by *Dv*, MmHM: hydrogenotrophic methanogenesis by *Mm*.


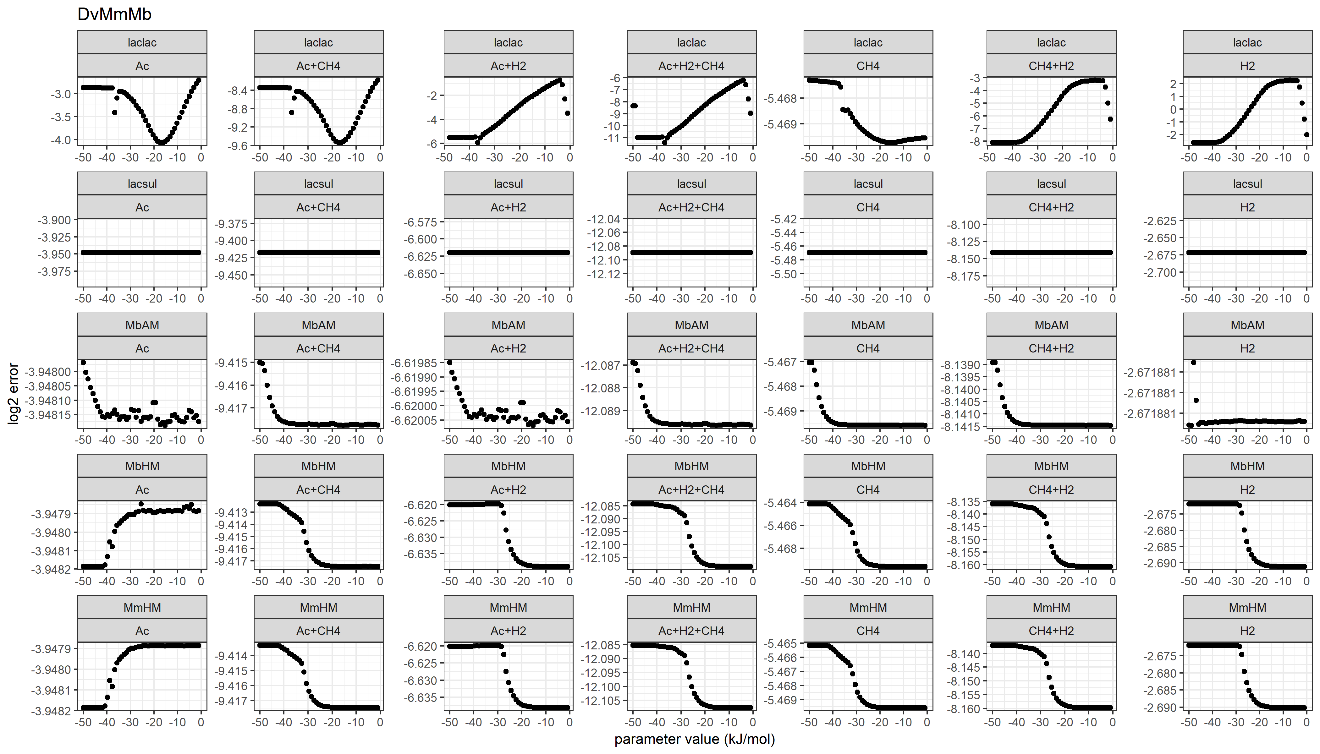


**Figure S4:** Log of the sum of squared differences (error) between the experimentally observed variable(s) and the model prediction. Results are shown for the *DvMmMb* triculture as a function of the value of the *ΔG_min_* parameter (kJ/mol) of various pathways and using specific experimental dataset (as indicated on each tile). Notation used for pathways is; laclac: lactate fermentation by *Dv*, lacsul: lactate respiration on sulfate by *Dv*, MbAM: acetoclastic methanogenesis by *Mb*, MbHM: hydrogenotrophic methanogenesis by *Mb*, MmHM: hydrogenotrophic methanogenesis by *Mm*.


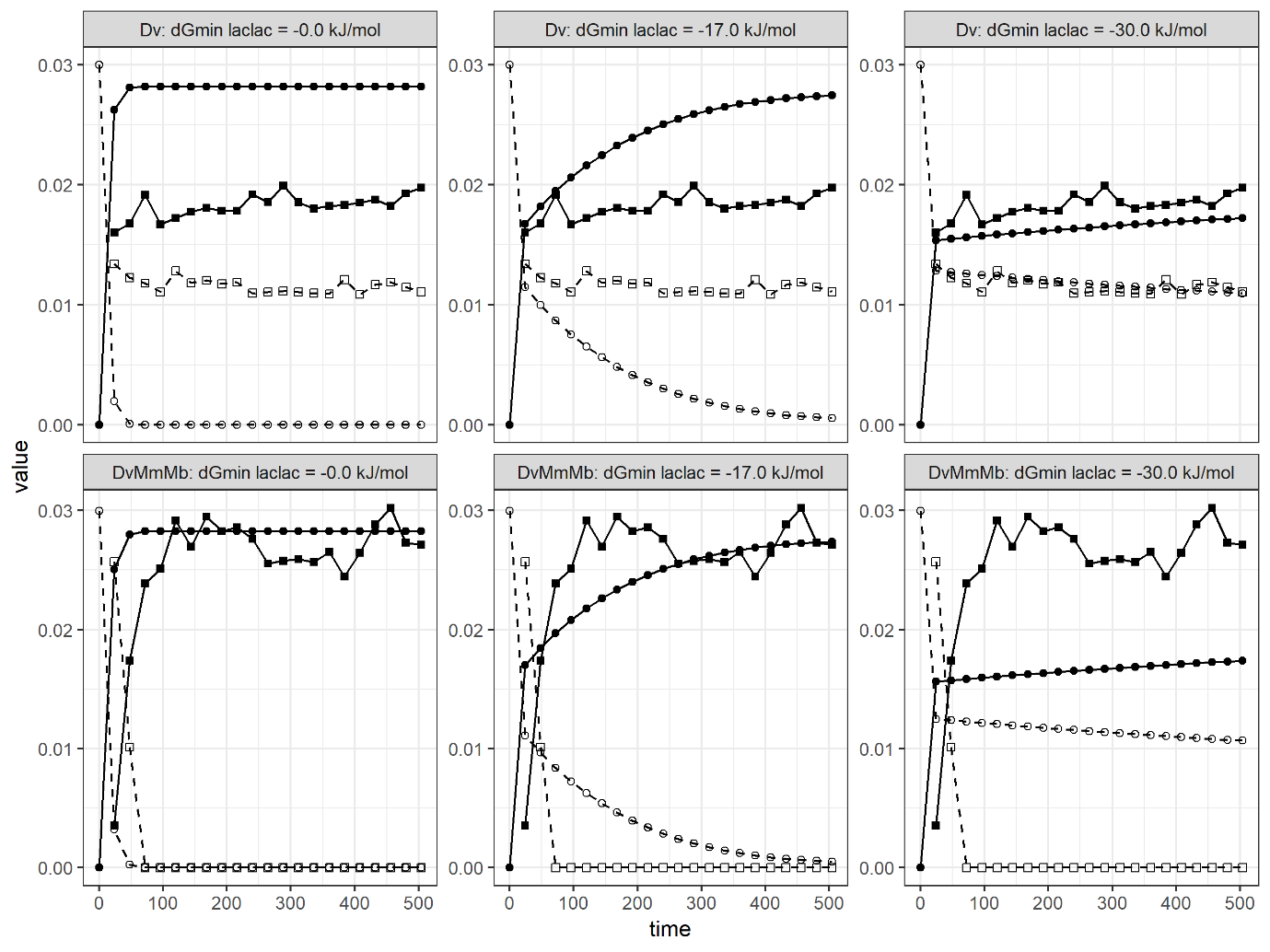


**Figure S5:** Concentration (mol/L) of acetate (solid line and filled shapes) and lactate (dashed line and empty shapes) over time (h) as measured in experiments (square) vs. simulated by the model (circle). Each set of panels on the two rows correspond to a culture; *Dv* (top) and *DvMmMb* (bottom). Each panel column corresponds to results of simulations done with a different value for the Gibbs energy threshold of the lactate fermentation pathway (“laclac”) as indicated on panel title.


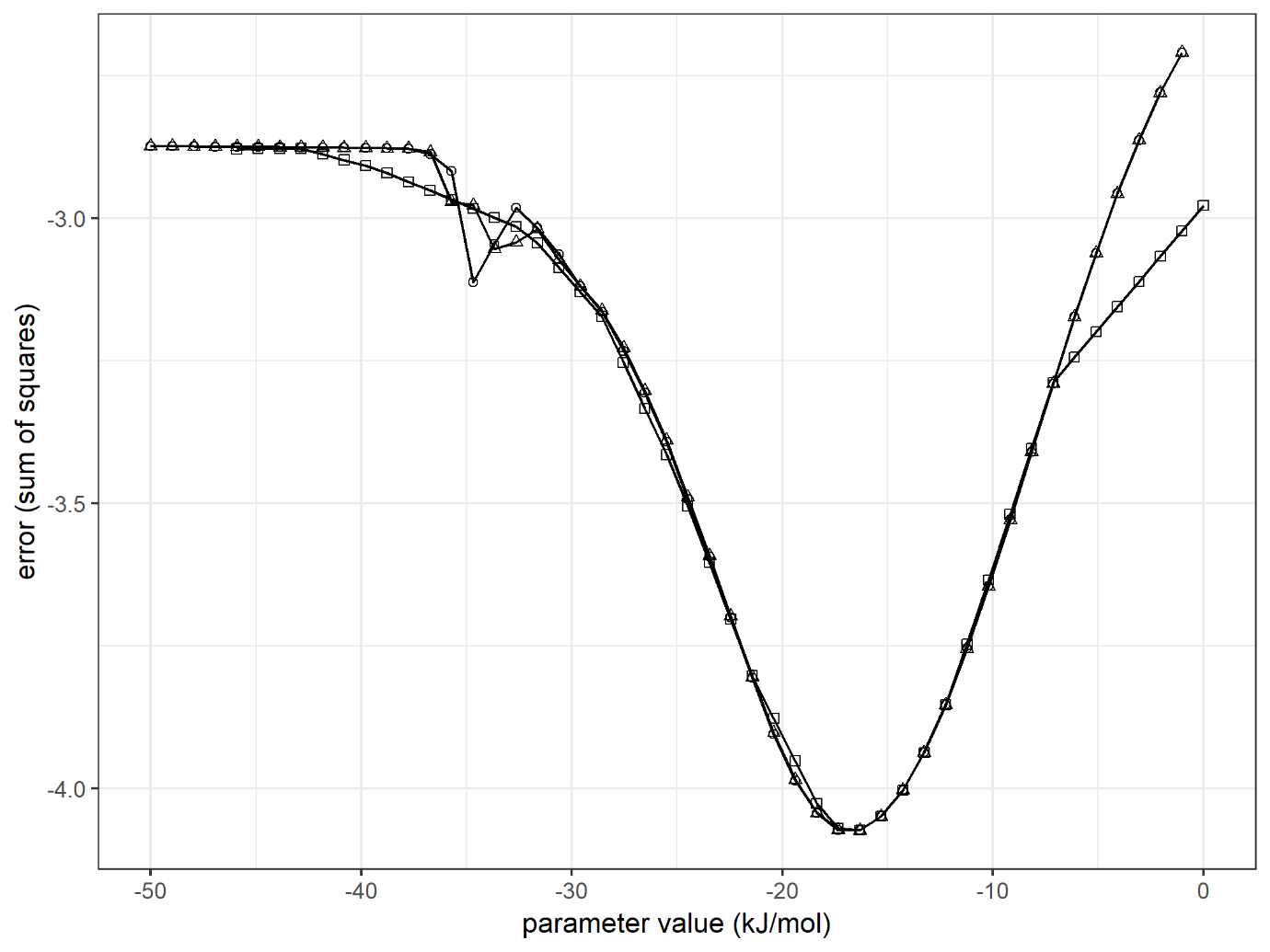


**Figure S6:** Sum of squared differences (error) between the experimentally observed acetate concentration and the model prediction in the *DvMmMb* triculture, as a function of the value of the *ΔG_min_* parameter for the lactate fermentation pathway. The three different curves correspond to different settings for the decay coefficient *k_d_* of each of the populations; all populations having the same decay coefficient, as in the main simulations of this manuscript (square), *Mb*’s and *Mm*’s decay coefficient being increased by a 20% factor (circles) and *Mb*’s and *Mm*’s decay coefficient being decreased by a 20% factor (triangles).

**References**

1. Noguera DR, Brusseau G a, Rittmann BE, Stahl D a. A Unified Model Describing the Role of Hydrogen in the Growth of Desulfovibrio vulgaris under Different Environmental Conditions. *Biotechnol Bioeng* 1998; **59**: 732–746.

2. Robinson JA, Tiedje JM. Competition between sulfate-reducing and methanogenic bacteria for H2 under resting and growing conditions. *Arch Microbiol* 1984; **137**: 26–32.

3. Westermann P, Ahring BK, Mah RA. Temperature Compensation in Methanosarcina barkeri by Modulation of Hydrogen and Acetate Affinity. 1989; **55**: 1262–1266.

4. Hoover SR, Porges N. Assimilation of Dairy Wastes by Activated Sludge : II . The Equation of Synthesis and Rate of Oxygen Utilization. *Sewage Ind Waste* 1952; **24**: 306–312.
